# SARS-CoV2 variants differentially impact on the plasma metabolome

**DOI:** 10.1007/s11306-025-02238-y

**Published:** 2025-04-05

**Authors:** Tina Kramaric, Onn Shaun Thein, Dhruv Parekh, Aaron Scott, Andrine Vangberg, Manfred Beckmann, Helen Phillips, David Thickett, Luis A. J. Mur

**Affiliations:** 1 Department of Life Sciences, Aberystwyth University, Penglais Campus, Aberystwyth, SY23 3DA UK; 2 Acute Care Research Group, Institute of Inflammation and Ageing, University of Birmingham, Birmingham, B15 2TT UK; 3 Birmingham Biomedical Research Centre, Institute of Translational Medicine, National Institute for Health and Care Research (NIHR), Birmingham, UK

**Keywords:** COVID19, SARS-CoV2 variants, Phospholipids, Ganglioside GD1a, Dihydroxyvitamin D3

## Abstract

**Introduction:**

Infection with severe acute respiratory syndrome-coronavirus-2 (SARS-CoV-2) leads to COVID19 disease and caused a worldwide pandemic in 2019. Since the first wave of infections, there has been significant antigenic shifts, leading to the emergence of new variants. Today, infections have shifted away from the severe, fatal infection seen in 2019.

**Objective:**

This study aimed to assess how the plasma metabolomes from patients varied with infection with different strains and could reflect disease severity.

**Methods:**

Patients with COVID19 not requiring intensive care were recruited between January 2021 and May 2022 from the Queen Elizabeth Hospital Birmingham; 33 patients with alpha, 13 delta and 14 omicron variants. These were compared to 26 age matched contemporaneously recruited controls. Plasma samples were extracted into chloroform/methanol/water (1:2.5/1 v/v) and assessed by flow injection electrospray mass spectrometry (FIE-MS) using an Exactive Orbitrap mass spectrometer. Derived data were assessed using the R based MetaboAnalyst platform.

**Results:**

Plasma metabolomes from COVID19 patients were clearly different from controls. Metabolite variation could be related to infection with different SARS-CoV2 variants. Variant showed different levels of some phospholipids, ganglioside GD1a and a dihydroxyvitamin D3 derivative. Correlations of the plasma metabolomes indicated negative correlations between selected phospholipids and the levels of C-reactive protein, creatinine, neutrophil and D-dimer.

**Conclusion:**

The plasma metabolomes of COVID19 patients show changes, particularly in phospholipids, which could reflect disease severity and SARS-CoV2 variant infection.

**Supplementary Information:**

The online version contains supplementary material available at 10.1007/s11306-025-02238-y.

## Introduction

The severe acute respiratory syndrome-coronavirus-2 (SARS-CoV-2) causes a lower respiratory tract infection through droplet/ aerosol spread between individuals. This leads to COVID19 disease which has a wide range of clinical phenotypes from the symptomless to severe infection needing ventilation support. Severe infection can be fatal and, as of 2023, is the cause of 14.83 million excess deaths globally (Msemburi et al., 2023) Mortality rates are highest in the elderly due to comorbidities such as cardiovascular and lung disease, hypertension, and diabetes (Sanyaolu et al., 2020; Sapey et al., 2020).

The SARS-CoV-2 genome encodes four structural (S [spike], E [envelope], M [membrane] and N [nucleocapsid]) and six accessory (3a, 6, 7a, 7b, 8, and 9b) proteins. The S protein is a key component of the SARS-CoV2 proteome which allows infection. The S protein has three subunits, a single-pass membrane anchor, an intracellular C-terminal tail, and the ectodomain. The ectodomain contains receptor-binding S1 and membrane-fusion S2 subunits (Mercurio et al., 2021). The S1 subunit interacts with the host angiotensin-converting enzyme 2 (ACE2) receptor which is most abundant in the upper respiratory tract mucosa, whilst the S2 subunit fuses the host and the viral membranes to aid viral entry (Mahmoud et al., 2020). Following initial ACE2 binding, the S1 and S2 subunits are cleaved by transmembrane protease serine 2 (TMPRSS2) (Takeda, 2022). This triggers fusion of the viral and host cell membranes, releasing positive sense viral RNA into the host cell (Jackson et al., 2022).

Global efforts have focused on identifying genomic variants of SARS-CoV2 which can evade host defences and reduce the effectiveness of vaccination strategies. Following Phylogenetic Assignment of Named Global Outbreak (Pango) designations the initial SARS-CoV2 represented the A type genome from which the B type evolved. The B type was the source of the variants of concern (VOC) during the COVID19 pandemic, i.e. B.1.1.7 (Alpha), B.1.351 (Beta), B.1.617/B.1.617.2 (Delta), P.1 (Gamma), and B.1.1.529 (Omicron) (Rambaut et al., 2020). The alpha variant is characterized by multiple spike protein mutations and showed increased transmissibility and disease severity compared to wild type (WT) SARS-CoV-2 virus. The beta variant emerged in South Africa and is characterized by 17 critical mutations in the S, E and N proteins and polyprotein encoding RNAs (ORFs 1a, 1b and 3a) and three deletions the N5 loop of the S protein. These mutations allowed increased transmissibility and also immune escape properties (Tegally et al., 2021). The gamma variant was first detected in Brazil to become initially predominant in South America and has 17 mutations including three in the S protein (Faria et al., 2021). The delta variant emerged in India and showed increased transmissibility and many subtypes were identified with common mutations in the spike protein (Rahman et al., 2021). The omicron variant emerged in southern Africa in late 2021 and showed high transmissibility and immune escape after full vaccination but exhibited reduced less severe disease compared to the delta variant (Sarkar et al., 2023). The continual evolution of SARS-CoV-2 leading to new variants which, even if they no longer constitute a public health emergency of international concern, still represent a continuing challenge. For example, between January and August 2024, COVID19 was listed as the cause of death in nearly 6000 death certificates in the UK (UKHSA, 2024). Therefore, there is the need for an ongoing development of treatments and effective vaccines. Such efforts need to be informed by an understanding of how infection mechanisms and symptom developments can change with each variant.

Systemic changes to inflammatory molecules can lead to changes in cytokine release and consequently inflammation. Circulating pro-inflammatory cytokines such as interleukin 6 (IL-6), Vascular endothelial growth factor (VEGF), soluble tumour necrosis factor receptor-1 (sTNFR1), granulocyte-macrophage colony-stimulating factor (GM-CSF) and myeloperoxidase (MPO) are different in COVID19 patients. Changes to the metabolome in COVID19 patients may be partially responsible for the poor outcomes seen compared to community-acquired pneumonia (CAP) patients with similar National Early Warning Score (NEWS) admissions figures. Therefore, metabolomic profiling has a role in COVID19 research and inform clinical practice. Saliva metabolomics may help predict COVID19 progression, recovery, and reflect changes of the underlying mechanisms causing temporary loss of taste (Costa Dos Santos Junior et al., 2020). Plasma could be used to detect biomarkers which were related to a major shift between mild and moderate COVID19 where inflammatory events were predominant (Su et al., 2020). Systemic analysis of serum metabolome changes correlated the inflammation-related ornithine cycle-related metabolites with the ‘cytokine storms’ observed in severe COVID19 (Li et al., 2021). Metabolomics could also be used to assess full recovery as patients in the respiratory recovery phase, who were testing negative, had abnormal metabolite profiles (Lodge et al., 2021).

Considering the metabolomic responses to discrete SARS-CoV2 variants, these have only been indirectly examined in serum samples from patients across the two epidemiological waves in 2021 (Lewis et al., 2022). Each wave produced distinctive changes in lipids (triacylglycerol [TG] (22:1_32:5), TG (18:0_36:3), glycolithocholic acid (GLCA) and some amino acids (glutamic acid and aspartic acid), which could reflect difference in inflammatory responses. However, there are no studies which have compared the impact of discrete SARS-CoV2 variants using untargeted metabolomics. Here, we used metabolomic approaches to assess changes in plasma from patients infected with three distinct SARS-CoV-2 variants to clinical markers of disease severity.

## Materials and methods

### Ethical statement and patient recruitment

Patients were recruited from the Queen Elizabeth Hospital Birmingham (QEHB), UK in accordance with ethics REC ref: 19/WA/0299 and 20/WA/0092. Enrolled patients had a positive point of care (antigen) COVID19 test on admission, or a positive polymerase chain reaction (PCR) COVID19 test result. SARS CoV2 Variants were identified by sequencing. The demographic details of the patients are listed in Table 1.

**Table 1:** Demographics, biochemical and clinical data from recruited patients (shading is added to ease reading)

	All COVID19	Alpha	Delta	Omicron
	*n* = 87	*n* = 41	*n* = 32	*n* = 14
**Male: Female**	48:39	26:15	17:15	5:9
**White: Non-white**	66:21	31:10	22:10	13:1
**Mortality (n=)**	17	10	7	0
**Age (years with range)**	70.4	71.5	72	82
	(57.0–84.3)	(58.0–84.0)	(51.8–83.0)	(72.5–93.8)
**Vaccinations (n=)**				
0	18	0	17	1
1	1	0	0	1
2	10	0	10	0
2 + booster	16	0	4	12
**Comorbidities (n=)**				
Cardiovascular	28	12	9	7
Respiratory	7	1	5	1
Endocrine	39	16	17	6
Hypertension	40	19	13	8
Chronic Kidney Disease	9	2	4	3
Other	50	24	16	10
**Clinical Assessments**				
White Cell Count (WCC) (x10^9^/L) with range	10.3	8.2	6.7	10.1
	(6.1–11.8)	(6.3–12.0)	(5.0–9.8)	(8.0–13.7)
Neutrophils (x10^9^/L) with range	7.4	6.4	5.2	8.8
	(4.3–8.8)	(4.4–8.6)	(3.6–8.7)	(6.4–12.1)
C-reactive protein (CRP) (mg/L) with range	111	103	81	35.5
(an inflammatory biomarker)	(43.3–160.3)	(63.0–165.0)	(41.0–336.0)	(24.5–59.8)
Neutrophil to lymphocyte ratio (NLR) with range	9.4	5.4	6.1	12.9
	(4.1–13.3)	(3.8–10.8)	(3.1–21.3)	(6.8–17.4)
High Sensitivity Troponin I (ng/L) with range	123.3	14.5	8	22
(an indicator of cardiac damage)	(7.5–36.5)	(5.0–31.3)	(5.3–282.0)	(22.0–26.0)
D-dimer (ng/mL) with range	2005.6	382	493	1365.5
(a fibrin degradation product)	(252.3–837.0)	(218.0–829.5)	(241.3–833.0)	(374.8–2496.0)
Ferritin (ug/L) with range	1714	1082	353.5 (	179
	(285.3–1489.8)	(428.3–1525.0)	203.3–1752.0)	(133.0–207.5)
Vitamin D (nmol/L) with range	49.1	35.6	37.8	51.9
	(24.6–62.9)	(23.0–51.8)	(23.3–62.3)	(26.2–64.9)
Dexamethasone treatment (n=)	67	38	19	10
Worst National Early Warning Score (NEWS) 2 with range	6	6	6	4
(degree of patient illness score)	(4.0–7.0)	(5.0–7.0)	(3.0–12.0)	(4.0–7.8)
4 C Mortality Score with range	11.3	12	11	13
(predicts in-hospital mortality)	(9.0–14.0)	(9.0–14.0)	(5.8–13.2)	(10.3–14.0)
quick Sepsis-related Organ Failure Assessment (qSOFA) score with range	1	1	1	1
	(1.0–1.0)	(1.0–1.5)	(1.0–1.0)	(1.0–1.0)
CURB-65 score with range	2	2	2	2
(clinical prediction of mortality in community-acquired pneumonia)	(1.0–3.0)	(1.0–3.0)	(2.0–3.0)	(2.0–3.0)
**Length of stay (days)**	6	5.5	5	8
	(3.0–12.0)	(3.0–12.0)	(3.0–11.5)	(5.0–11.8)

### Metabolomic sample Preparation

Samples were dispatched on dry ice from the University of Birmingham (UK) to Aberystwyth University (UK), on dry ice. On arrival, samples were immediately moved to a −80^o^C freezer. Plasma samples were centrifuged for 5 min at 4 °C and 21,000 x *g* after which 200 µL of each sample was aliquoted into labelled 2 mL microcentrifuge tubes. A total of 1520 µL of a HPLC grade methanol: chloroform (4:1) solution was added to a micro-insert in a glass vial after which 100 µL of the adjusted plasma sample was added and the vial was sealed by cap crimping. A pooled “master mix” sample containing equal aliquots from each sample was used as a quality control to compensate for any drift in signal intensity.

### Flow infusion electrospray ionization high resolution mass spectrometry

Extracted metabolites were analysed by flow infusion electrospray ionization high resolution mass spectrometry (FIE-MSHRMS) in the High-Resolution Metabolomics Laboratory (Aberystwyth University). The samples were profiled using an Exactive Orbitrap (ThermoFinnigan, San Jose CA) mass spectrometer. Samples (20 µL volume) were injected by an autosampler into a flow of 100 µL/min methanol/water (70:30, v/v). Pre-mixed ultra-pure H_2_O (18.2 Ω) and HPLC grade MeOH (Fisher Scientific) at a ratio of 7:3 or a flow solvent (mobile phase) were used to deliver 20 µL of the injected sample to the electrospray ionisation (ESI) source. ESI source parameters were set based on manufacturer’s recommendations. Metabolite fingerprints were generated in both positive and negative ionisation mode. Ion intensities were acquired between *m/z* 55 and 1200 for 3.5 min in profiling mode at a resolution setting of 280,000 (at m/z 200) resulting in 3 (± 1) ppm mass accuracy. Spectral binning approach used BinneR which also eliminated anomalous single scan *m/z* events, the averaged of spectra across the infusion profile. The modal accurate *m/z* was then extracted for each bin (Finch et al., 2022). Data were log_10_-transformed and Pareto scaled to transformed to normality and used for statistical analysis performed by MetaboAnalyst 5.0 (Peng et al., 2022). The non-transformed data are presented in Table S1.

### Statistical analysis

Principal component analyses (PCA), partial least squares discriminant analysis (PLS-DA) and its variable importance in projection (VIP), receiver operating characteristic (ROC) curves, t- tests, ANOVA, Pearson’s correlations, and heat maps of hierarchical cluster analyses (HCA) were generated using custom scripts in R and MetaboAnalyst (Xia et al., 2012). PLS-DA models were validated using training and test sets, where the results were accepted when Q^2^ = > 0.6 and Leave-one-out cross-validation (LOOCV) accuracy > 0.6. Pearson’s correlations were tested for significance by Student’s *t* test, corrected for false discovery rate (FDR). The diagnostic potential of discrete metabolites was assessed based on the ROC curves (Fawcett, 2006). Derived AUC values of ≥ 0.70 were considered to be of potential clinical relevance and this was used to select the key metabolites. BinneR also accurate *m/z* to metabolites based on the MZedDB ionisation ‘rules’ (Draper et al., 2009; Finch et al., 2022). The *m/z* were tentatively identified based on the Human Metabolome Database (https://hmdb.ca/) which included consideration of isotope and different forms of ionisation and based upon both a maximum error of 5 ppm.

## Results

### Metabolomic comparisons between COVID19 and healthy patients

Plasma samples were assessed using the FIE-MS in negative and positive ionisation modes and the derived spectra were analysed using multivariate approaches. Initial assessments compared COVID19 and controls samples. No discrimination was made between the severity of COVID19 symptoms.

**Fig. 1:**
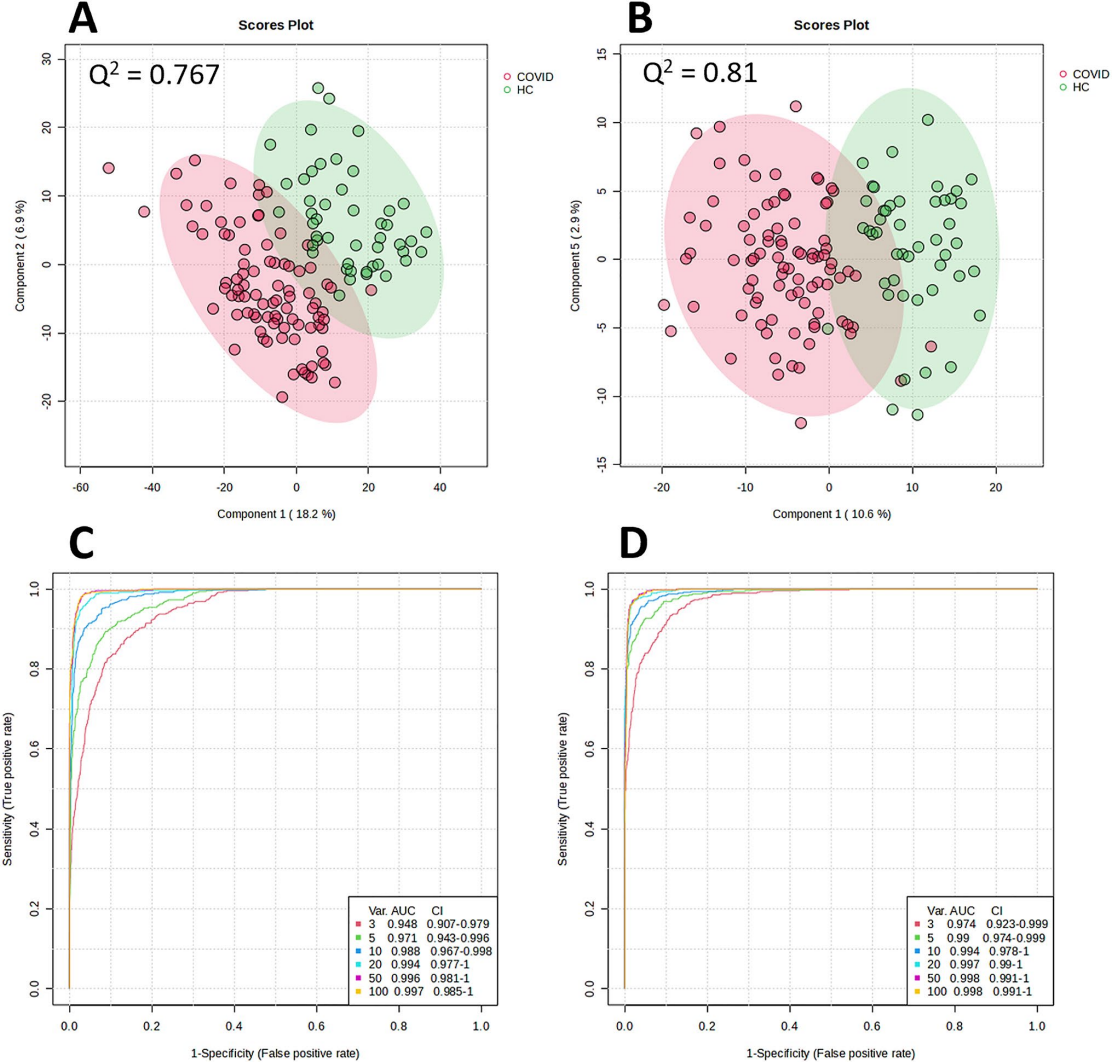
PLSDA and Receiver operating characteristic (ROC) curves of negative (**A** and **C**) and positive (**B** and **D**) ionisations for the COVID-19 and healthy control groups. Q² values are given for each PLSDA as a measure of the predictive relevance of each model

PLS-DA showed clear separation of the groups along component 1 (Fig. 1A, B). The major sources of variation were identified by *t*-tests correcting for FDR, and ROC curve assessments (Table 2). Multivariant ROC curves assessments suggested an AUC value for the top five discriminatory metabolites of 0.971 (97.1%) for negative ionisation (Fig. 1C), and 0.99 (99%) for positive ionisation (Fig. 1D). COVID19 patients showed changes in the levels of some glycerophospholipids, an acylcarnitine, sphingolipid, a myo-inositol signalling component, a monosaccharide, a steroid and a prostaglandin (Table 2).

**Table 2:** The major sources of variation between the plasma of COVID19 patients and healthy controls with tentative identifications

m/z	Ionisation	AUC	*p*-value	Log2 Fold Change*	ID	Chemical class
385.2961	Negative	0.89507	5.21 × 1019	2.9401	20-oxo-heneicosanoic acid	Fatty acyl
412.91	Negative	0.90027	2.16 × 1019	2.6757	D-myo-inositol-trisphosphate	Signal molecule
286.1155	Negative	0.87041	1.84 × 1017	3.4105	(2E)-hexenedioylcarnitine	acylcarnitine
490.2914	Positive	0.96917	8.23 × 1023	−1.7787	PC (0:0/14:0)	Glycerophospholipids [GP]
440.8085	Positive	0.91405	3.12 × 1021	3.5299	Cer(d18:1/22:0(2OH))	Sphingolipid
373.0465	Positive	0.94246	8.50 × 1021	3.066	3-hydroxy-estra-1,3,5(10),6,8-pentaen-17-one	Steroid
320.9883	Positive	0.92324	1.25 × 1020	3.0381	1-[(2R,3R,4 S,5R)−3,4-Dihydroxy-5-(hydroxymethyl) oxolan-2-yl] oxypyrimidin-2-on	Monosaccharide
441.0962	Positive	0.92759	1.84 × 1020	2.9173	Prostaglandin H2	Eicosanoids
800.5203	Positive	0.94608	2.08 × 1020	−1.2744	PS or PE phospholipid	Glycerophospholipids [GP]
752.5225	Positive	0.9317	9.53 × 1020	−0.83399	PS or PA phospholipid	Glycerophospholipids [GP]

Based on data corrected for statistical normality and false discovery rates

### Metabolomic comparisons between SARS-CoV2 variants in COVID19 patients

**Fig. 2:**
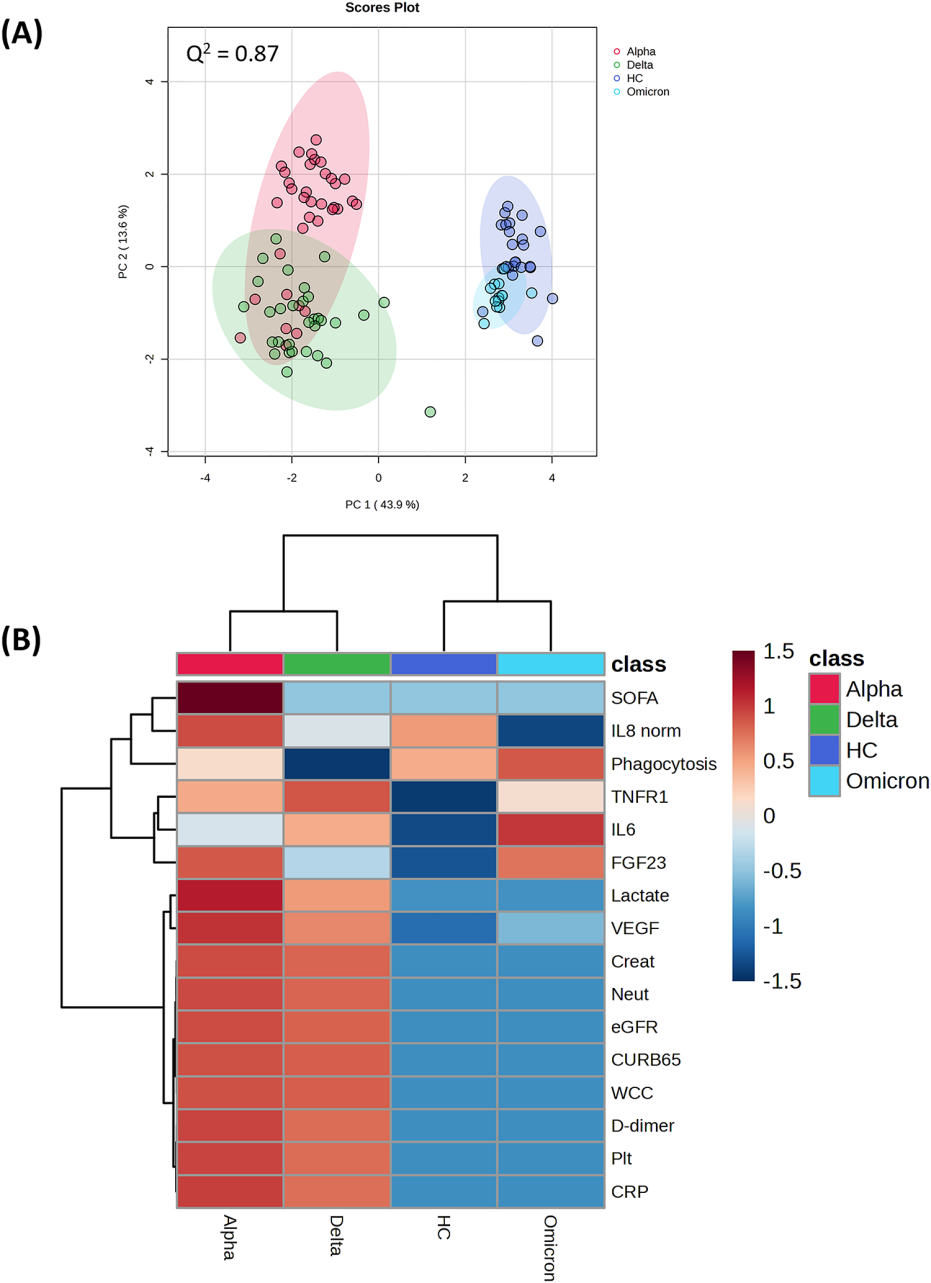
**A** PCA and **B** HCA showing separation of the COVID-19 variants and healthy controls (HC) using clinical data. The Q² values for the PLSDA is a measure of the predictive relevance of the model. SOFA = sequential organ failure assessment; IL8 norm = normalised interleukin 8 levels; VEGF = Vascular endothelial growth factor; Creat = creatinine; Neu = neutrophil cell levels; eGFR = estimated Glomerular Filtration Rate; CURB65 = a clinical prediction rule for predicting mortality in community-acquired pneumonia; WCC = white cell counts; D-dimer = dimer that is a fibrin degradation product; Plt = platelet count; CRP = C-Reactive protein levels, FGF23 = Fibroblast growth factor 23; IL6 = interleukin 6 levels; soluble tumour necrosis factor receptor 1 (TNFR1)

To begin to relate metabolomic change to specific variants, we first examined the variation in the clinical features seen with each COVID19 group. The clinical features which significantly (*P* = < 0.05) differed between infection with each SARS-CoV2 were identified by ANOVA (Table 3). This identified neutrophil counts (Neu) as the most significant clinical feature that differed between the infections with different variants. PLS-DA of the provided clinical data showed clear differences with between the variants, with alpha and delta variants clustering together but the omicron variant and healthy controls forming a separate cluster (Fig. 2A). The major source of variation across PC1, appeared include C-reactive protein (CRP), vascular endothelial growth factor (VEGF), neutrophil counts (Neu), D-dimer, creatinine (Creat), and soluble tumour necrosis factor receptor 1 (TNFR1). These were compared using a heat map (Fig. 2B). The clinical variables readily separated the alpha-delta variant from the omicron-HC groups. The alpha group appeared to have much higher sequential organ failure assessment (SOFA) scores than the delta infected patients. Whilst patients with omicron infections had many similarities with the HC group, the former showed increased in IL6, TNFR1 and FGF23.

**Fig. 3:**
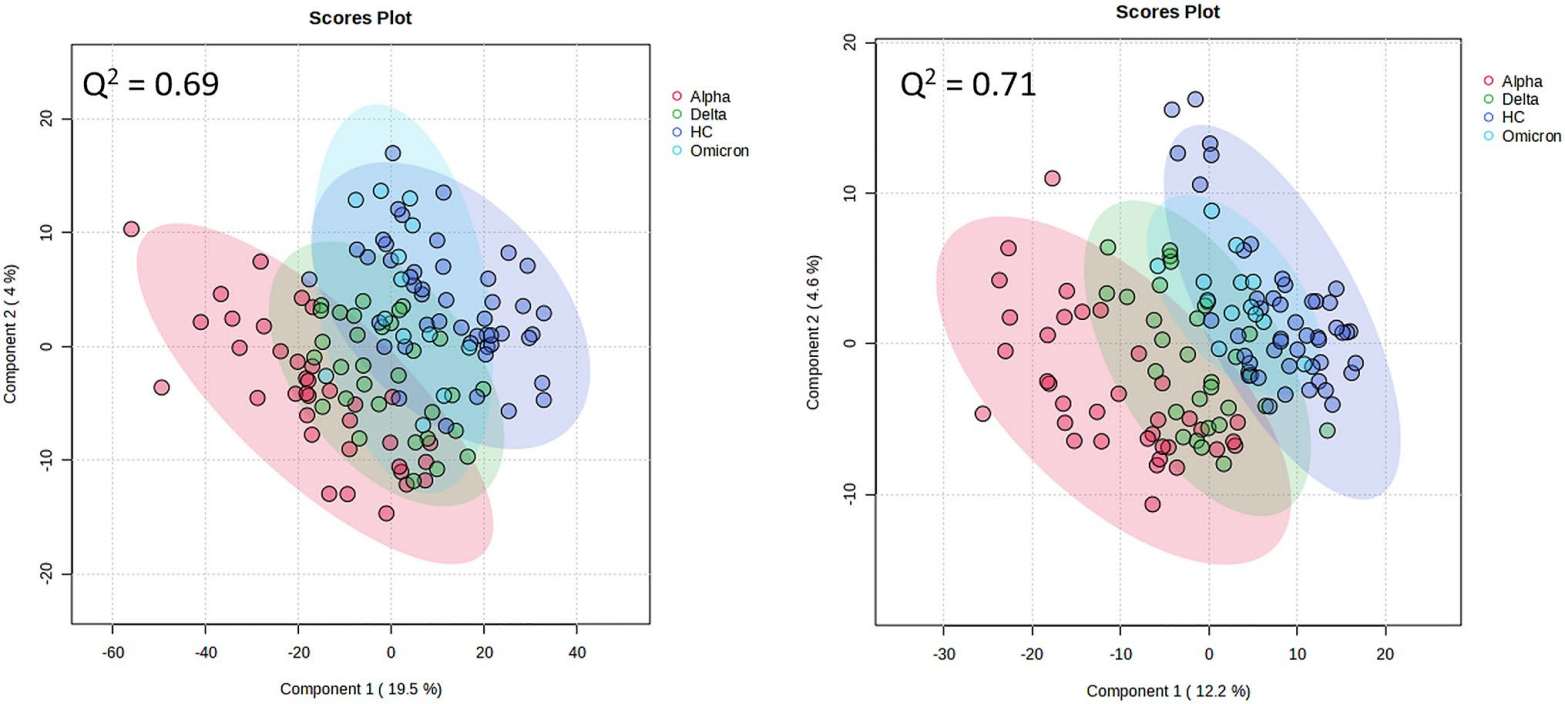
PLSDA of negative (**A**) and positive (**B**) ionisations for the COVID-19 variant (alpha, delta, omicron) and healthy control (HC) groups. Q² values are given for each PLSDA as a measure of the predictive relevance of each model

**Fig. 4:**
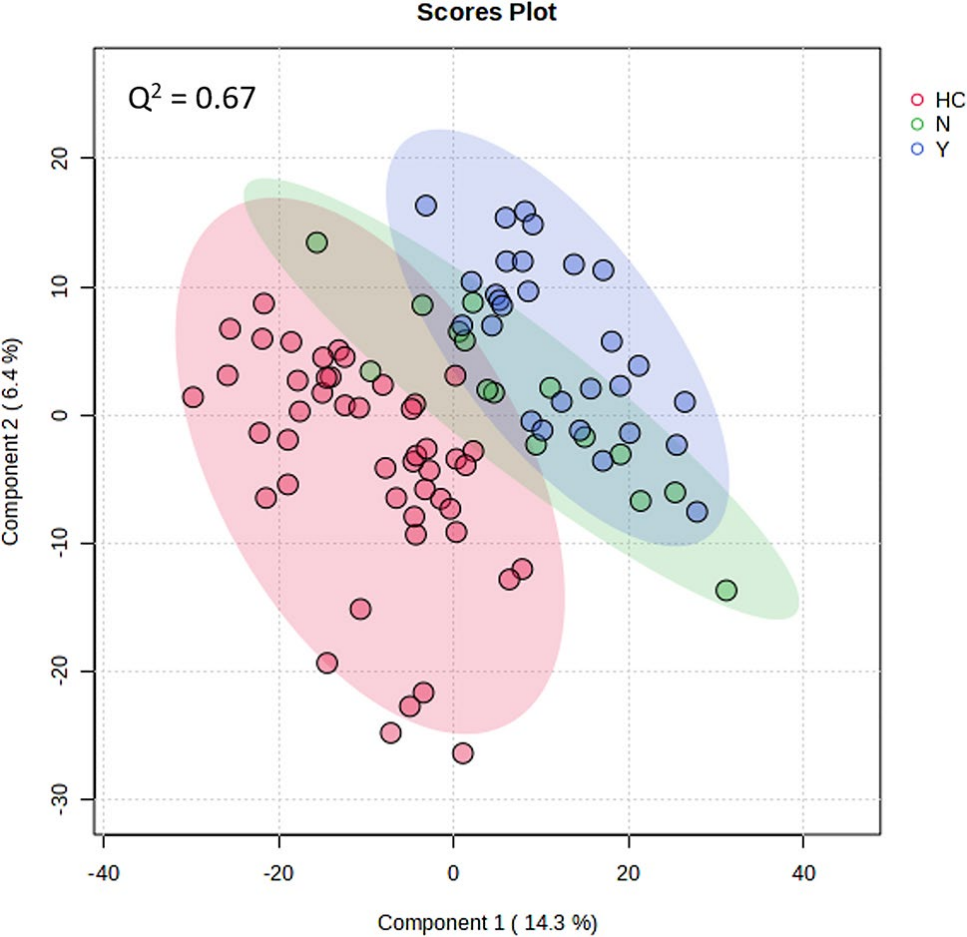
PLSDA of negative ionisations of plasma from patients which had been vaccinated (Y), not vaccinated (N) and healthy controls (HC). Q² values are given for each PLSDA as a measure of the predictive relevance of each model

Metabolomic profiles were then classified based on SARS-CoV2 variant (alpha, delta, omicron) and HC samples (Fig. 3A, B). The metabolite profiles derived following either positive or negative ionisation were most distinctive for the alpha SARS-CoV2 variant samples. Plasma from patients infected with the omicron variant appeared to be indistinguishable from healthy controls. Samples from patients with delta variants, appeared to show some difference from healthy controls and the alpha variant. We next considered if patient vaccination status could be a confounding source of variation in the data (Fig. 4). When samples were stratified based on vaccination status, no difference was seen in the plasma metabolomes. The metabolomes of both vaccinated and non-vaccinated sample differed from healthy controls. Sex and race did not affect the separation of samples seen in Fig. 3.

**Table 3:** Clinical features significantly differentiating between SARS-CoV2 variants

Clinical Feature	*P*-value	FDR
Neutrophil counts	6.02 × 10^35^	1.14 × 10^33^
Platelet (Plt) count	1.14 × 10^32^	1.08 × 10^31^
eGFR	2.76 × 10^32^	1.53 × 10^31^
Creatinine concentrations	3.21 × 10^32^	1.53 × 10^31^
C-Reactve Protein	1.11 × 10^29^	4.23 × 10^29^
WCC	1.45 × 10^27^	4.60 × 10^27^
qSOFA	1.12 × 10^21^	3.03 × 10^21^
Lactate	5.27 × 10^21^	1.25 × 10^20^
R fold change	7.47 × 10^17^	1.58 × 10^16^
CURB65	1.49 × 10^14^	2.83 × 10^14^
G fold change	2.61 × 10^14^	4.51 × 10^14^
D-dimer	2.03 × 10^12^	3.22 × 10^12^
IL6	1.63 × 10^8^	2.39 × 10^8^
Phagocytosis	2.76 × 10^8^	3.74 × 10^8^
TNFR1	6.92 × 10^7^	8.76 × 10^7^
VEGF	1.64 × 10^6^	1.95 × 10^6^
IL8	9.00 × 10^5^	1.01 × 10^5^
FGF23	0.002195	0.002317

eGFR = estimated Glomerular Filtration Rate (eGFR); WCC = white cell count; qSOFA = quick Sepsis-related Organ Failure Assessment (qSOFA) score; IL6 = Interleukin 6, TNFR1 = Tumour necrosis factor receptor 1 (TNFR1), VEGF = Vascular endothelial growth factor; IL8 = Interleukin-8; FGF23 = Fibroblast growth factor 23

**Fig. 5:**
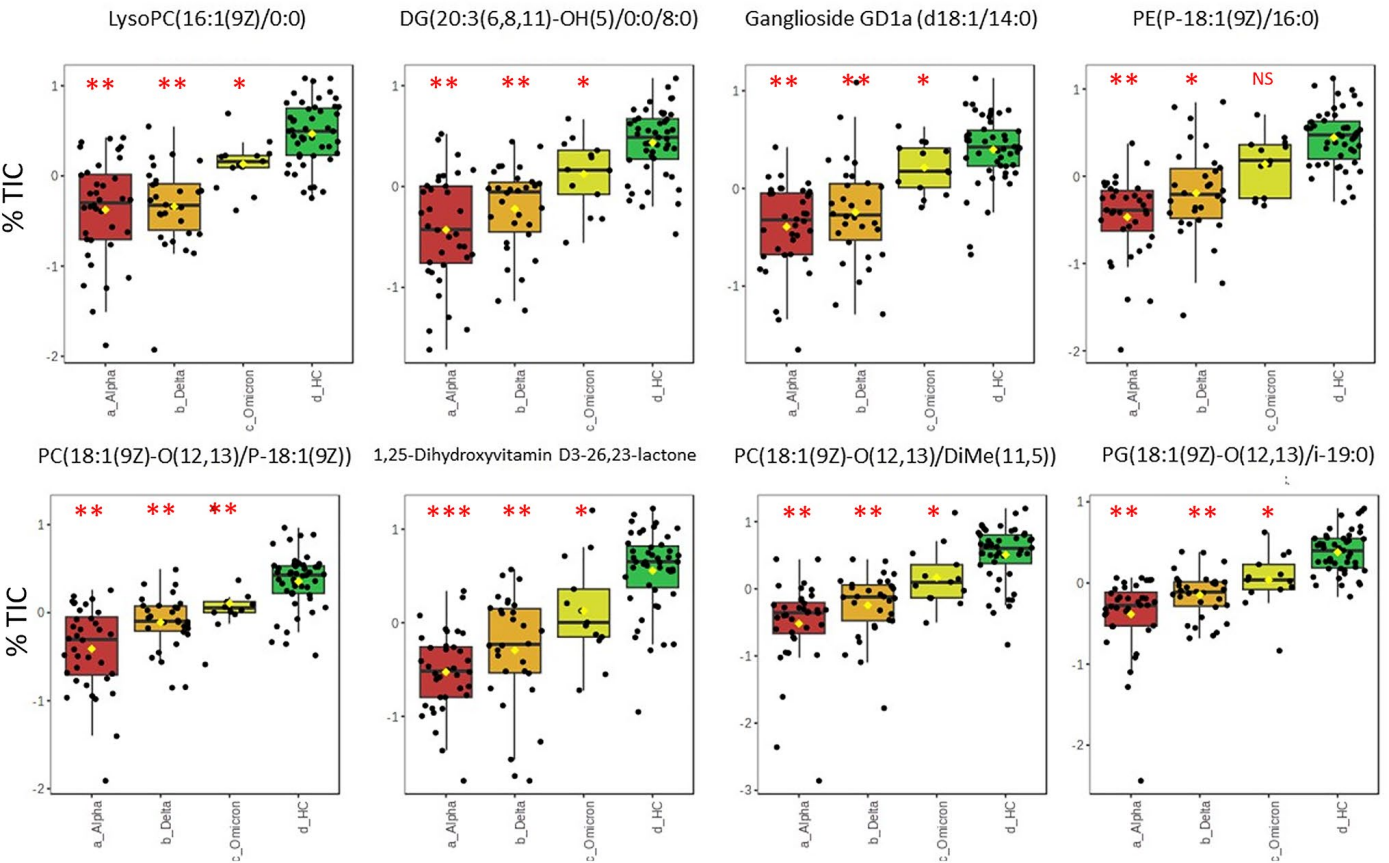
Box and whisker plots showing sources of variations between the SARS-CoV2 variants and the healthy controls. Values are shown as percentage total ion count (% TIC). Significant differences to the healthy controls (HC) are indicated as *** = *P <* 0.05, ** = *P <* 0.01, * = *P <* 0.05 and NS = Non-significant based on ANOVA with *post hoc* Fisher LSD. Metabolites are tentatively identified

ANOVAs correcting for FDR were used to identify the major sources of variation in the metabolomes from different variants. None of the variables targeted in the COVID19 vs. control comparison (Fig. 1) were specific to a particular SARS-CoV2 variant. Metabolites which were changing with variants, appeared to be significantly (*P* < 0.05) reduced in plasma from alpha variant infection compared to healthy controls (Fig. 5). These changes appeared to be in fatty acids and phospholipid (glycerophospholipids and a lysophosphatidylcholine [LysoPC]) changes. There were also significant reductions in a vitamin D3 metabolite and ganglioside GD1a compared to controls.

### Correlating metabolomic changes with SARS-CoV2 variants to clinical features

**Fig. 6:**
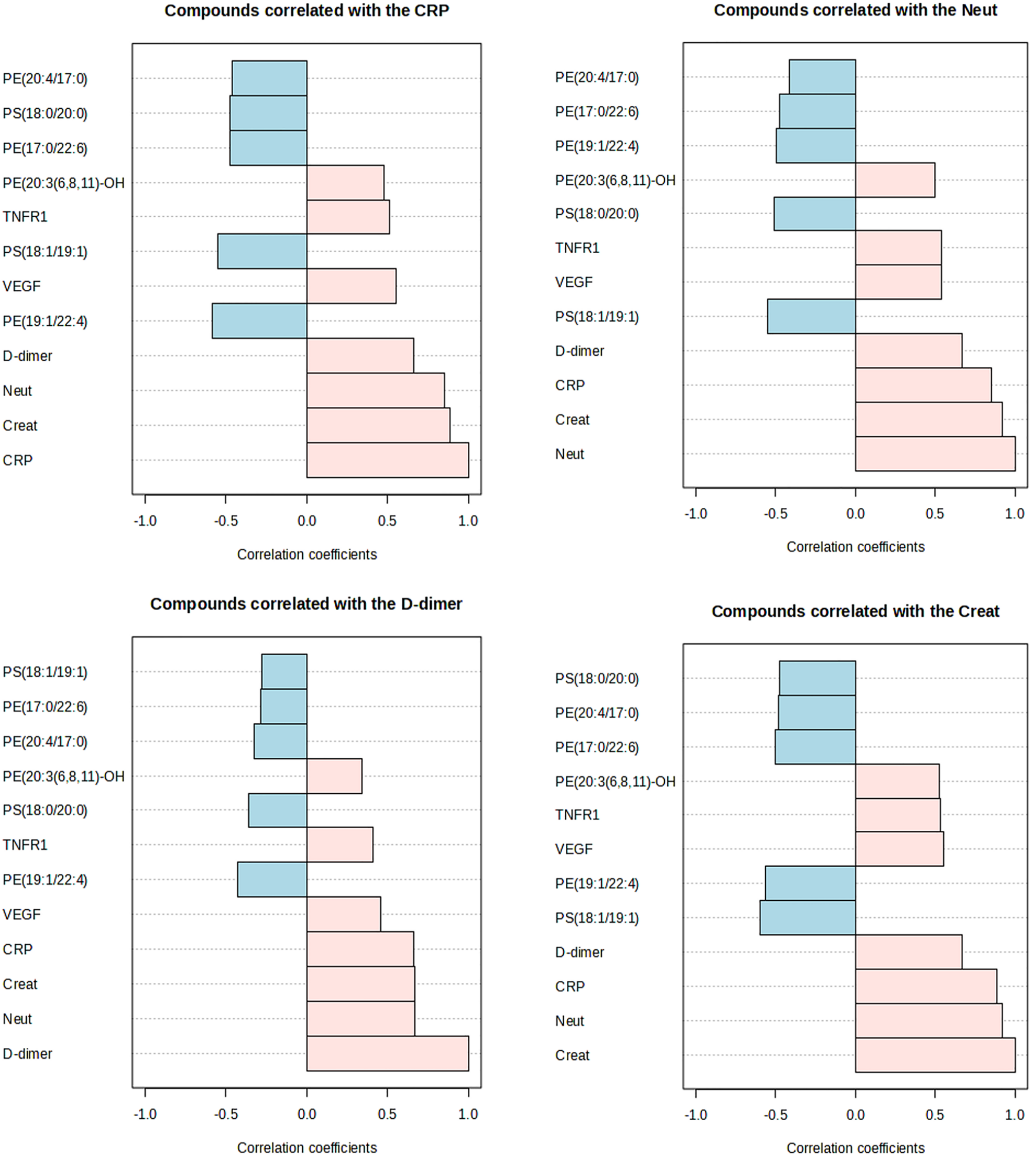
Pearson’s Correlation Coefficients of metabolite changes with CRP, creatinine (Creat), Neutrophils (Neut), and D-dimer levels: CRP = C-Reactive protein; soluble tumour necrosis factor receptor 1 (TNFR1), VEGF = ascular endothelial growth factor; Phospholipid classes: PE = Glycero phosphoethanolamines; PS = Glycerophosphoserines

Next, we attempted to correlate metabolomic changes in the plasma with clinical features of SARS-CoV2 infection based on Pearson correlation coefficients. Within the clinical data, CRP levels showed positively correlated with creatinine, D-dimer, neutrophil count, sTNFR1 and VEGF levels. Examining variables which differed with different types of variants (Fig. 3), significant (*P = <* 0.05) positive correlations between CRF, Neut, D-dimer and Creat were seen with the phosphatidylethanolamine PE (19:1/22:4) and PE (20:3(6,8,11)-OH) with other targeted PEs and phosphatidylserine (PS) showing a negative correlation (Fig. 6).

## Discussion

In this study, we first assessed whether our population of COVID19 patients would show the typical metabolomic changes compared to a healthy population. We observed clear differences in COVID19 patients compared to controls which we could link to metabolite changes that had been seen in other studies. Thus, although our analyses yielded tentative identifications, such outcomes suggest the value of our observations.

The formation of acylcarnitines occurs when fatty acids are transported across the mitochondrial membranes to drive energy metabolism. The formation of a range of acylcarnitines has been noted in the serum of COVID19 patients (Martínez-Gómez et al., 2022) and also in our study (Table 2). These changes suggest increases in lipid processing COVID19 patients to meet a high energetic demand (Páez-Franco et al., 2021) as well as the activation of inflammatory pathways (Herrera-Van Oostdam et al., 2021). These effects are likely to be reflected in our observed reduction in the levels of some glycerophospholipids in COVID19 patients as well as an increase in a proinflammatory eicosanoid (Table 2). Such inflammatory events could also be reflected in the increases seen in a ceramide sphingolipid (Cer(d18:1/22:0(2OH) in COVID19 patients (Table 2) which has also been noted by Khodadoust (2021) which could tribute to viral damage in lung tissue (Khan et al., 2023). Inositol trisphosphate (IP3) is derived from the hydrolysis of phosphatidylinositol 4,5-bisphosphate and is a potent signal that can trigger the release of calcium from the endoplasmic reticulum. IP3 effects are classically associated with cell proliferation but within the context of SARS-CoV2 infections could be contributing to mucin overproduction in the airway epithelium (Khan et al., 2021).

We next examined the metabolomic changes which could be associated with different SARS-CoV2 variants. Phospholipid depletion in the plasma of COVID19 patients is associated with the elevated cytokine storm which leads to alveolar epithelial cell injury and alveolar collapse on expiration (Hussain et al., 2021). Phospholipids were also decreased in the plasma of deceased COVID19 patients compared to healthy controls, and COVID19 patients who died also showed a significant decrease in phospholipid levels compared to survivors. This is likely to reflect the impact of oxidative stress as well as increases in phospholipid and free polyunsaturated fatty processing enzymes, phospholipase A2, lipoxygenases and cyclooxygenases (Žarković et al., 2022). Infections with the omicron variant appeared to result in a lesser suppression of phospholipids (Fig. 4) which would suggest lower levels of lipid processing and oxidative stress. The relevance of phospholipids is further reflected when correlating the metabolomes with clinical features. Significant negative correlations were found between phospholipid levels which were reduced with disease severity and the CRP and D-dimer values which correspondingly increased (Fig. 2B). Other studies have observed an increase of CRP and D-dimer and a decrease of phospholipids in the plasma COVID19 patients at the acute infection stage compared to healthy controls (Abdalla et al., 2020). Thus, the relative levels of phospholipid levels are associated with the severity of inflammation, hypoxia in COVID19 patients (Dei Cas et al., 2021), which we here relate to SARS-CoV2 variants.

Another discriminatory lipid metabolite of interest was ganglioside GD1a, where the sphingolipid is linked to a neutral tetrasaccharide core with sialic acid residues. Ganglioside GD1a has been linked to autoimmune disorders, for example, Guillain-Barré syndrome (GBS) in nervous tissue. COVID19 has been suggested to be a trigger for GBS. With SARS-CoV2 infection there is an increase in GD1a autoantibody (Valaparla et al., 2024) and this could the targeting of the cognate ganglioside resulting in the reduced levels observed in our metabolomic study. GD1a levels appeared to return to control levels in patients with omicron infections, which would suggest a link between this ganglioside and the extent of disease severity.

One potential pathway to increased systemic inflammation is though changes to neutrophil function. Neutrophils are a key early inflammatory mediator in infection, and we observed that neutrophil counts were the most significant clinical feature that varied with the different variants with levels being the highest with infections with the alpha SARS-CoV2 variant (Table 3). One aspect of neutrophil function is related to linoleic acid metabolism which is linked to adenosine triphosphate (ATP) production. These regulate the formation of neutrophil extracellular traps (NETs) in cattle during the peripartum period (Alarcón et al., 2020). High levels of NET formation are thought to be related to poor outcomes and high rates of immunothrombosis, which may account for the high incidence of thromboembolic disease in COVID19 patients. Whilst linoleic acid was not one of the metabolites targeted by our assessments, the lowering of certain glycerophospholipids (Fig. 4) and negative correlations with established proinflammatory biomarkers (Fig. 5) may indicate their processing to release such as linoleic acid.

Another metabolite that appeared to be lowered with disease severity, one important example was a 25-hydroxyvitamin D derivative. A deficiency in 25-hydroxyvitamin D is linked to poorer outcomes for sepsis patients in intensive care units (Singh et al., 2022) and increases the risk of a 30-day mortality in patients with severe sepsis or septic shock (Rech et al., 2014). This aligns with the suggested effect of lower levels of vitamin D linked to more severe COVID19 symptoms (Pereira et al., 2022). Our previous study also suggested that vitamin D deficiencies led to an increases risk of seroconversion for COVID19 in UK healthcare workers (Faniyi et al., 2021).

There are some confounding factors that could have influenced our observations, only some of which we could address. Most obviously, the vaccination programme that was implemented in the UK after the first wave of alpha variant could have influenced the metabolome. Other studies have noted metabolomic shifts in response to the tuberculosis vaccine Bacillus Calmette-Guérin (BCG) (Koeken et al. 2022) or between high-risk responders and non-responders to influenza vaccination (Rodrick et al., 2023). Most relevant, assessment of high and low responders to the Pfizer BioNTech (BNT162b2) COVID19 vaccine showed changes in such as phenylalanine, histidine, methylhistidine and glutamine (Dagla et al., 2022). This study also showed changes in ceramides, including Cer(d18:1/22:0) that we also observed. Whilst we had no information on whether our patient population were high or low responders to vaccination with the Oxford–AstraZeneca or Pfizer COVID-19 vaccines, we did not note any differences in the plasma metabolomes that could be linked to vaccination status. Further, most of the plasma metabolites that we observed changing with SARS-CoV2 variant, had been previously reported to change with vaccination (Ali et al., 2022).

Another confounding factor was the impact of COVID19 treatment with dexamethasone. Following on early trials (e.g. RECOVERY Collaborative Group et al., 2021; Ghanei et al., 2021), dexamethasone became established as an important strategy in the treatment of COVID19 (WHO Rapid Evidence Appraisal for COVID-19 Therapies (REACT) Working Group, Sterne et al., 2020). Treatment with such corticosteroid drugs has been shown to affect the metabolome in COVID19 patients, for example, altering cortisol or bile acid levels (Spick et al., 2022), or hydroxyestradiol, carnitine, lysine and prostaglandin G2 levels (Mwangi et al., 2023). Whilst most of our COVID19 patients infected with any variant were treated with dexamethasone, it is possible that differential patient responses to this corticosteroid, may have influenced our results.

## Conclusions

Taken together our metabolomic analyses have suggested distinctive changes in plasma for infections with each SARS-CoV2 variant. There appeared to be no discrete changes seen with a given variant, but differences could be linked to difference in their relative elicitation of core pathophysiological events associated with COVID19, for example, inflammation. Additionally, the more severe COVID19 appears to be related to greater losses in glycerophospholipid content which could reflect increased bioenergetic demands. Whilst our observations require confirmation via larger studies that include targeted identification of metabolites, these observations could suggest therapeutic interventions to suppress core COVID19 metabolomic effects.

## Supplementary Information

Below is the link to the electronic supplementary material.

Supplementary material 1 (XLSX 7088.0 kb)

## Data Availability

All metabolomic data are available as supplementary data - Table S1. ELISA results are available upon request.
